# The role and perception of the caregiver in a specialized pediatric palliative care center in medicine preparation and administration: a survey study

**DOI:** 10.1186/s13052-024-01809-4

**Published:** 2024-11-06

**Authors:** Fernando Baratiri, Chiara Zanella, Barbara Roverato, Daniele Mengato, Laura Camuffo, Lisa Pivato, Irene Avagnina, Irene Maghini, Antuan Divisic, Francesca Rusalen, Caterina Agosto, Francesca Venturini, Franca Benini, Anna Zanin

**Affiliations:** 1 Palliative Care and Pain Service, Department of Women’s and Children’s Health, University of Padua, via Giustiniani 3, Padua, 35128 Italy; 2 Department of Women’s and Children’s Health, University of Padua, Padua, Italy; 3 Hospital Pharmacy Department, Azienda Ospedale - Università Padova, Padua, Italy

**Keywords:** Pediatric palliative care, Caregiver, Therapy preparation, Medication burden, Administration errors

## Abstract

**Background:**

In pediatric palliative care, the main caregiver is primarily responsible for managing pharmaceutical therapies. Few data are available regarding the influence of this burden on quality of life in terms of time, concerns as well as a considerable risk of administration errors and adverse effects. This study aims to investigate how caregivers prepared and administrated medication, including errors and associated expectations, to identify improvement interventions.

**Methods:**

Between October 2022 and March 2023, a descriptive single-center survey study was carried out in the tertiary care pediatric palliative center of the Padova University Hospital. Participants were the caregivers of the patients followed by our center up to 23 years old, receiving at least one drug daily and who cannot self-administer their therapy. The questionnaire consisted of 18 multiple-choice and semi-closed questions, grouped into 4 main topics: therapy preparation, therapy administration, administration errors and therapy assessment.

**Results:**

A total of 100 caregivers responded to the survey. Mothers represented the main caregiver (91%). The prevalence of polypharmacy was 67% across the patients. 52% of caregivers handled prescriptions at least three times per day and for 32% it took to prepare them more than 5 min each time. Only 59% reported to have been trained for preparing and administrating drugs. 14% reported having made at least a drug administration error due to the tiredness or the complexity of therapeutic regimens in the preceding three months. Nearly one caregiver out of three felt their child was using too many drugs. 73% positively welcomed the possibility of having clinical pharmacist-led counseling.

**Conclusions:**

Many caregivers of pediatric palliative care patients frequently have trouble planning, preparing and delivering pharmacological therapy to their children. Attempting to simplify medication regimens, choosing formulations that are simpler to administer and measure, investing in improved caregiver training, talking about therapies with carers, and involving clinical pharmacists to clarify their doubts could be all potential strategies to improve this condition and reduce their burden.

**Supplementary Information:**

The online version contains supplementary material available at 10.1186/s13052-024-01809-4.

## Introduction

In the context of Pediatric Palliative Care (PPC), caregivers are mostly responsible for the preparation and administration of pharmacological therapy with a significant impact on quality of life (QoL) in terms of time and costs, which are often not adequately recognized and quantified. Patients followed by PPC often are exposed to complex medication regimens (CMR) or polypharmacy to relieve their symptoms and achieve the best QoL [1]. Caregivers may lack appropriate and standardized training for the correct preparation and administration of pharmacological therapy and this aspect contributes to the risk of making errors with potential harm for the patients [2]. Many fundamental drugs are not commercially available in children-tailored formulations or doses. Thus, caregivers can be forced to manipulate prescribed medications to meet the needs of the patient [3, 4]. Such manipulations (such as tablet crushing, capsule opening, dilution and administration via feeding tubes) could be performed multiple times per day and, when not specified in the summary of product characteristics (SmPC), are considerable as an off-label drug use and can lead to an alteration of the administered dose and a modification of the drug bioavailability [5, 6]. The clinical impact for patients is the increasing risk of adverse effects and poor symptom control. Moreover, manipulating medication before administration seems to have a negative influence on treatment adherence [7] which is critical to guarantee the best symptom control. The impact of chronic therapy on caregivers of children with long-term conditions such as asthma, cystic fibrosis, diabetes, attention deficit, chronic intestinal diseases, or transplant recipients has been investigated [8–15]. The most common concerns expressed by caregivers include the appropriateness of the ongoing therapy and its potential side effects, medication administration and intake at school, interference with social activities, the rigidity of the treatment regimen and treatment adherence. Supporting caregivers is complex and needs of team working involving nurses, physicians, social workers and pharmacists. These latter could be useful in resolving caregivers’ concerns and doubts. It has been well defined in adult patients, it provides beneficial services such as increasing therapeutic compliance and adherence, reducing the likelihood of adverse effects or administration errors and decreasing hospital readmissions [16–20]. This might lead to many benefits also for pediatric patients and their caregivers, but unfortunately, the literature about this population is poor and there are no studies in the setting of PPC.

### Aims

This study aims to investigate the experiences of caregivers regarding the manipulation, preparation and administration of pharmacological therapy, including any administration errors, the associated expectations to identify possible improvement actions.

## Materials and methods

### Study design and setting

A descriptive single-center survey study was conducted between October 2022 to March 2023 in the Pediatric Palliative Care Center of Veneto Region of Padova, Italy.

### Study population and recruitment

The study involved caregivers of patients followed by our PPC center up to 23 years old, receiving at least one current medication and who could not administer the therapy by themselves. Caregivers who provided non-continuous and/or episodic (meaning less than twice per week) patient care or if their children did not meet the inclusion criteria were excluded from the study. We decided to include patients up to 23 years old because they are referred to our center until this limit of age. Patients usually from 18 years of age start the transition phase from pediatric to adult care but, due to the complexity of the process, this may last many years [21]. Moreover, many of their physical and clinical characteristics (ex. low weight, cognitive impairment, need of constant care) make them closer to pediatric standards of care.

### Questionnaire description

No validated questionnaires were available in the literature to assess and investigate the management of pharmacotherapy by caregivers in patients followed by PPC. Thus, we designed an 18-questions survey, in Italian, specifically for this study. A multidisciplinary team consisting of palliative care physicians, pediatricians, nurses and clinical pharmacists with proven experience in the field designed and developed the questionnaire in two phases. The first phase consisted of collecting the main concerns and difficulties from the caregivers through informal interviews. The second one consisted of setting up a preliminary test of the questionnaire and administering it to a small group of caregivers before validating and extending it to the whole group of selected caregivers.

The questionnaire consisted of both multiple-choice and semi-closed questions, grouped into 4 main topics: therapy preparation, therapy administration, administration errors, and therapy assessment.

The initial questions (1–6) assess therapy preparation challenges, including the impact on daily management and the need for training. Questions 7–12 focus on therapy administration, seeking insights on adherence and potential strategies for easier administration. The subsequent section (questions 13–14) aims to uncover recent administration errors and their causes. The final questions (15–18) evaluate therapy outcomes, caregiver burden, and the need for therapy adjustments in discussions with clinicians or pharmacists. The English version of the survey is provided as additional file 1.

### Data collection and analysis

Caregivers completed the questionnaire, which was administered by health care providers at the time of patients’ follow-up visits or hospital admissions or during scheduled telephone contacts, after appropriate information and signed consent.

Demographics and pharmacological data including information about patients’ baseline conditions (gender, age, main diagnostic category and “do not resuscitate” DNR order), principal symptoms and pharmacological therapies (number of drugs prescribed, dosage, frequency and route of administration) were collected from the electronic medical charts. All drugs were classified according to the Anatomical Therapeutic Chemical (ATC) classification.

Data about caregivers such as their profession, academic background or socio-economic parameters, were collected during the interview. To study a possible association between the risk of incurring a treatment error and the socio-cultural status of the caregiver, we decided to create a special score, the SCARES (Socio-Cultural Assessment for caregiver Risk of medication Error Score). This index takes into account the following aspects that could potentially expose a patient to possible errors: education below high school level, full-time job commitment, presence of a language barrier, single parent, need for night-time administration, patient with polypharmacotherapy, need to manipulate the drug before administration, congenital disease in therapy for more than ten years, age of the child under 5 years, inability to administer drugs via enteral tubes. One point was awarded for each of these 10 items, with a maximum total of 10 points per caregiver. This score was correlated, by means of Pearson correlation analysis for paired data, with the risk of error reported by the caregivers themselves. An r-value greater than 0.5 is regarded as a positive correlation between the two variables and is considered statistically significant at *p* < 0.05 [22]. The remaining data were analyzed using means ± SD for normally distributed variables, and medians with ranges or IQR for non-normally distributed data. Categorical variables were reported as frequencies and percentages, and analyzed with the χ2-test or Fisher’s exact test. Significance was set at *p* < 0.05.

Data were collected using an online survey tool (Google form) and subsequently cleaned and analyzed through an electronic spreadsheet (Microsoft Excel). The analysis was performed by multiple researchers independently to ensure reliability and validity.

### Ethical aspects

This study was conducted in accordance with Good Clinical Practice (GCP) using the guidance documents and practices offered by the International Conference on Harmonization and the European directives 2001/20/CE and ISO 14,155, and in agreement with the local regulations. The final protocol and its amendments were reviewed and approved by the local Ethical Committee (EC). Written informed consent was obtained from a parent and/or legal guardian.

## Results

### Patients’ characteristics

Of 307 patients followed by our Center at the time of the survey, 159 patients were identified as eligible for our study. Of these, 100 of their caregivers agreed to participate and a total of 100 patients were analyzed. Table 1 reports their baseline characteristics. The median age was 12 (IQR 5–16). The majority of patients were of Italian origin (*n* = 73, 73%). 93% have been followed for a congenital or perinatal-onset condition and only two patients had an oncological condition. The most common primary diagnostic categories were neurologic (*n* = 55, 55%) and musculoskeletal (*n* = 23, 23%). A total of 26 patients had the DNR order and three patients were in a terminal stage of their disease. Due to medical conditions and/or their age, 85 individuals could not self-feed and 62 of them had feeding tubes such as nasogastric (NG), gastrostomy (G) or jejunostomy (J) tubes.

**Table 1:** Patients’ characteristics

	*N* (%)	Mean (± SD)	Median (min-max)
Total population	100 (100%)		
Male	56 (56%)		
Female	44 (44%)		
Age (years)		10.8 (± 6.4)	12 (0–23)
Age category			
Infant (0–2 years)	13 (13%)		
Preschool (3–5 years)	13 (13%)		
School (6–11 years)	22 (22%)		
Adolescent (12–17 years)	34 (34%)		
Adult (> 18 years)	18 (18%)		
Congenital disease	93 (93%)		
Diagnostic category			
Cardiocirculatory	2 (2%)		
Genetic	4 (4%)		
Neurologic	55 (55%)		
Muscular-skeletal	23 (23%)		
Oncological	2 (2%)		
Respiratory	3 (3%)		
Other	11 (11%)		
Alimentation			
Oral	38 (38%)		
Feeding tubes	62 (62%)		
DNR^1^	26 (26%)		
Origin			
Italy	73 (73%)		
Eastern Europe	11 (11%)		
North Africa	10 (10%)		
Asia	4 (4%)		
Sub-saharan Africa	1 (1%)		
Central America	1 (1%)		

^1^ *DNR: do not resuscitate*

**Fig. 1:**
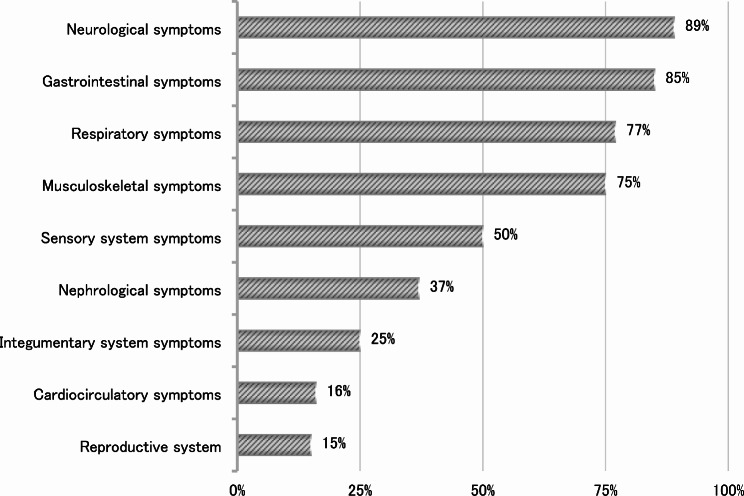
Prevalence of principal symptoms and needs according to different body systems

Figure 1 (TITLE: Prevalence of principal symptoms and needs according to different body systems) represents principal symptoms and needs prevalence across the population. Neurological, gastrointestinal and respiratory symptoms were the most prevalent. The most common symptoms reported were: cognitive impairment (*n* = 74, 74%), mobility impairment (*n* = 55, 55%) and seizures (*n* = 53, 53%) for neurological symptoms, dysphagia (*n* = 79, 79%), inadequate oral intake (*n* = 56, 56%) and constipation (*n* = 44, 44%) for gastrointestinal symptoms and excessive respiratory secretions (*n* = 46, 46%) and chronic respiratory failure (*n* = 45, 45%) for respiratory symptoms. We reported also 7 individuals with sexual needs. (See the table in additional file 2 for a detailed report of symptoms and needs.)

### Caregivers’ characteristics

Table 2 describes caregivers’ characteristics and their social-cultural aspects. 95% of patients had natural parents. The main caregiver was the mother in 91% of cases. No significant difference in education degree was found between fathers and mothers. However, mothers had a significantly higher unemployment rate. The average number of cohabitants in the family resulted in 4 ± 1.4 (median 4, IQR 3–5). 69% of patients had siblings, of these 43% were younger and 26% older.

**Table 2:** Socio-economic aspects of caregivers

	Whole cohort	Mother	Father	Other	*p*-value
Parenthood					
Natural parents	95 (95%)				
Adoptive/legal guardians	5 (5%)				
Marital status					
Married/living together	89 (89%)				
Divorced	7 (7%)				
Widowed	2 (2%)				
Unknown	2 (2%)				
Main caregiver		91 (91%)	4 (4%)	5 (5%)	**< 0.001**
Education					
College graduate		23 (23%)	17 (17%)		0.28
High school diploma		44 (44%)	42 (42%)		0.77
Middle school diploma		21 (21%)	22 (22%)		0.86
Elementary school diploma		1 (1%)	4 (4%)		0.17
Unknown		11 (11%)	15 (15%)		0.7
Employment					
Full-time job		22 (22%)	83 (83%)		**< 0.001**
Part-time job		19 (19%)	3 (3%)		**< 0.001**
Unemployed		59 (59%)	14 (14%)		**< 0.001**

### Pharmacotherapy

**Fig. 2:**
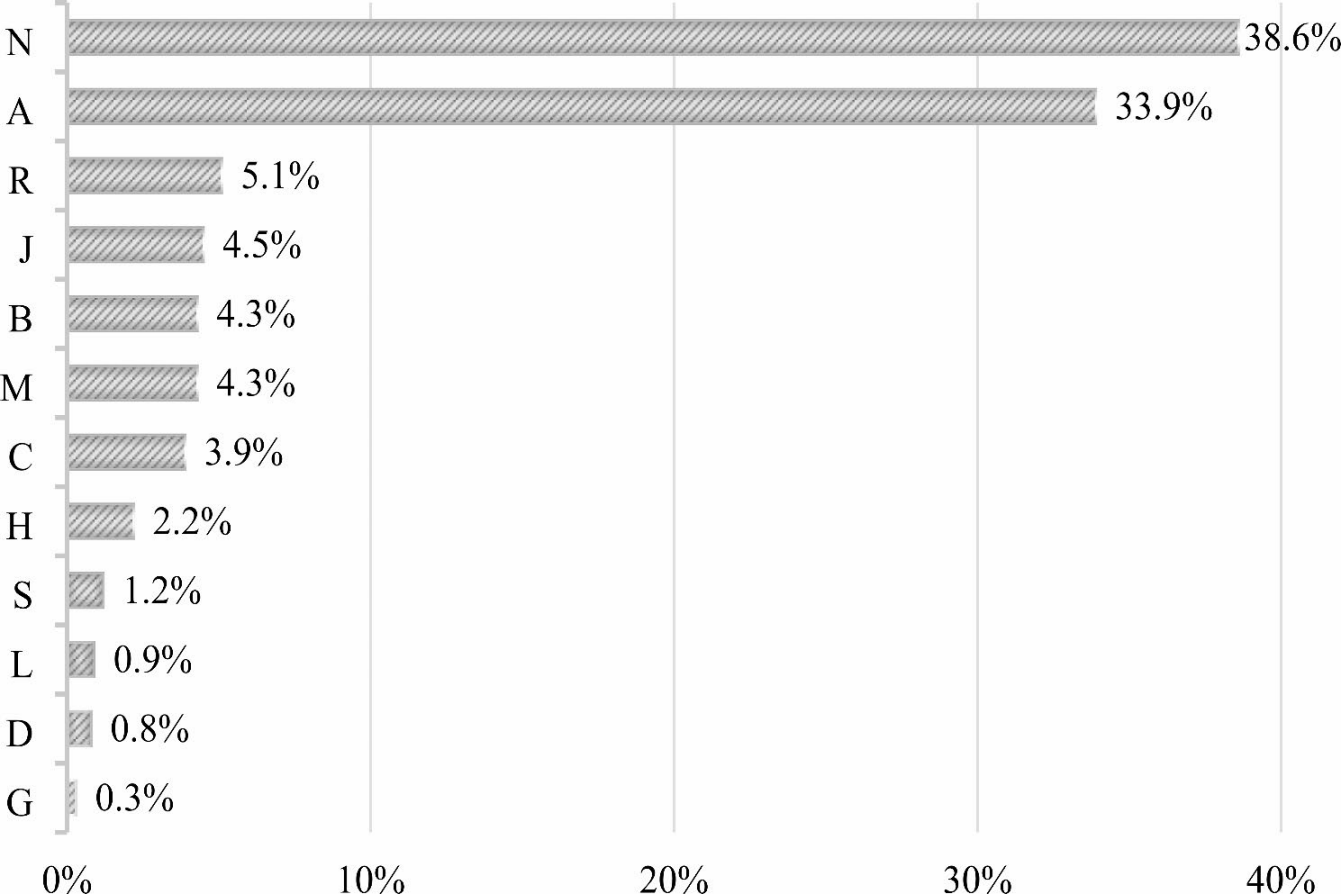
Prevalence of different drugs according to ATC classification; Legend “ATC: Anatomical Therapeutic Chemical (ATC) classification. Total drugs, n = 741. N: Nervous system (n = 286); A: Alimentary tract and metabolism (n = 251); R: Respiratory system (n = 38); J: Anti-infective for systemic use (n = 33); B: Blood and blood forming organs (n = 32); M: Musculoskeletal system (n = 32); C: Cardiovascular system (n = 29); H: Systemic hormonal preparations, excluding sex hormones and insulins (n = 16); S: Sensory organs (n = 9); L: Antineoplastic and immunomodulating agents (n = 7); D: Dermatological (n = 6); G: Genito urinary system and sex hormones (n = 2)”

Table 3 shows the prevalence of polypharmacy and details of patients’ therapies. 67% of patients were on polypharmacy defined as the current assumption of five or more different drugs. The median number of drugs prescribed for patients was 7 (IQR 4–11). The median number of total administrations per day was 10 (IQR 4–16), with a maximum of 35. Each treatment regimen included a median of 3 (IQR 2–4) ATC classes. A total of 741 drug prescriptions were analyzed. The most prevalent drug classes were represented by neurologic (*n* = 286, 38.6%), alimentary tract and metabolism (*n* = 251, 33.9%) and respiratory (*n* = 38, 5.1%). See Fig. 2 for more detail (TITLE: Prevalence of different drugs according to ATC classification). Oral and enteral assumptions were the most frequent routes of administration (*n* = 679, 91.6%). Other routes of administration as sublingual, rectal, transcutaneous, inhalation and intranasal account together for 55 prescriptions (7.5%) and parenteral administrations (intravenous, intramuscular) only 7 prescriptions (0.9%).

**Table 3:** Mean and median number of total drugs prescribed for patient, number of drugs as needed and their distribution and administration during the day

	*N* (%)	Mean (± SD)	Median (IQR)		Range
Polypharmacy	67/100 (67%)				
Total drugs		7.4 (± 4.5)		7 (4–11)	1–22
Scheduled drugs		6.4 (± 3.9)		6 (3.7-9)	1–20
“As needed” drugs		1.1 (± 1.3)		1 (0–2)	0–5
Daytime^1^ drug administrations		6.9 (± 4.7)		6 (3–10)	1–24
Night-time^2^ drug administrations		3.8 (± 3.2)		3.5 (1–6)	0–14
Total drug administrations		10.7 (± 7.6)		10 (4–16)	1–35
ATC^3^ drug classes		3.2 (± 1.4)		3 (2–4)	1–7

^*1*^ *Daytime = from 8:00 AM to 7:59 PM;* ^*2*^ *Night-time = from 8:00 PM to 7:59 AM;* ^*3*^ *ATC = Anatomical Therapeutic Chemical classification*

### Questionnaire responses

#### Medication preparation

10% of respondents reported difficulties in preparing therapies and 15% encountered challenges in accurately measuring the medication dose. The time required for therapy preparation varied among respondents, with 68% spending less than five minutes, 19% between 5 and 10 min, and 13% more than 10 min. Medication handling (tablet crushing, capsule opening or dilution and administration via feeding tubes) was widespread among responders (87%) and 52% of them needed to handle it at least 3 times per day. 59% of responders felt adequately trained for therapy preparation. 65% received training from our PPC center healthcare providers, 17% from the hospital healthcare providers, 12% from their pediatrician or general practitioner, 4% from their neurologist and one from a pharmacist.

#### Drug administration

43% percent of the caregivers reported difficulties in adhering to the time intervals between administrations. Oral and enteral have been reported as the preferred route of administration and they were considered well tolerated and more adapted to the patients’ clinical conditions. 75% of caregivers reported no difficulties in the child’s acceptance of the administered therapy. All cares of children with a feeding tube reported no difficulties or “rarely”. 43% mixed medications with soft foods or beverages (e.g. milk, fruit juices, sweetened drinks), to facilitate administration and to improve acceptance. 55% of them did it for each administration.

#### Administration errors

14% of caregivers reported at least an administration error in the previous 3 months. The tiredness (35.7%) and the complexity of the therapeutic regimens (28.6%) were reported as the principal causes. See Table 4.

**Table 4:** Responses about administration errors

	*N* (%)
Have you ever administered medications incorrectly in the past three months?	
Yes No I don’t remember	14% 83% 3%
What do you think was the cause of this error?	
I didn’t feel adequately trained in medication preparation I had too many administrations and got confused I was tired and inattentive Other Not reported	1/14 (7.1%) 4/14 (28.6%) 5/14 (35.7%) 2/14 (14.3%) 2/14 (14.3%)

#### Medication judgment

77% were satisfied with the current medication regime and the symptoms control achievement, although 41% thought their child takes too many medications “always” or “often”. 39% reported they wanted to discuss with medical staff their doubt about therapies. Lastly, 73% responded positively to the possibility of discussing the patient’s therapy with a clinical pharmacist.

The whole survey responses are provided as additional file 3.

#### SCARES score evaluation

**Fig. 3:**
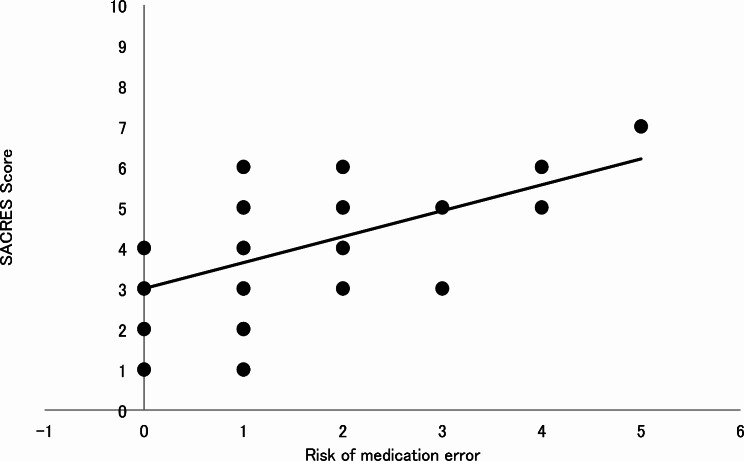
Correlation analysis between the SCARES score and the error risk

Figure 3 (TITLE: Correlation analysis between the SCARES score and the error risk) illustrates the correlation analysis between the SCARES score and the error risk. As depicted in the chart, despite being able to ascertain the score for only 62 caregivers, the correlation is positive with *r* = 0.53 (*p* < 0.05). The main represented items were: “need to manipulate the drug before administration” (52/62), “patient with polypharmacotherapy” (47/62) and “need for night-time administration” (42/62).

## Discussion

Despite the increasing attention on the prevalence of polypharmacy in the pediatric context due to the rising number of pediatric complex conditions [23–25] the impact on caregivers’ life of a complex drug administration and their daily management has not been deeply investigated. This is the reason why we wanted to collect the experiences of caregivers of patients of our PPC Center.

CMR are difficult to manage for clinicians because of the multiple possible interactions but also for caregivers who need specific and personalized training. To date, there are few dedicated tools to help clinicians and pharmacists to identify and measure the various aspects of the burden associated with medication complexity in the adult population [26, 27] and no data are available for pediatric palliative care patients. Only about half (59%) of our caregivers reported having had training in the use of their child’s drug therapy. The implementation of parental training should be renewed regularly to minimize errors in preparation or administration. The lack of many child-friendly formulations impacts negatively the time invested in medicine preparation with a reduction of QoL for caregivers. We estimated that most of the caregivers were investing around 3 h/week in preparing medicaments whereas half of them (43%) reported difficulties in maintaining the correct interval and had to do some manipulation by mixing the medications up with some food or beverages. It could lead to an unpredictable modification of the medication bioavailability [28] with a possible increase of adverse drug effects and, if administered via feeding tubes, obstruction and malfunctioning. All of these therapeutic schemes must be re-evaluated individually to understand the appropriateness and efficacy of the manipulations concerning the pharmaceutical form, as well as whether it is possible to lighten or bundle the administration strategy to facilitate the caregiver’s role. As suggested by Burlo et al. galenic compounds could be helpful for caregivers to simplify therapy administration, improving quality of life for patients and their families and possibly reducing administration error [29]. It’s also worth noting that 14% of parents acknowledged making mistakes in preparation or administration in the previous three months, implying that the true number of cases of failure when the caregiver was unaware might be greater. The major reasons cited were tiredness and load during therapy administration, as well as the complexities of the therapeutic regimens.

A post-hoc analysis was carried out trying to identify the items that could correlate with the risk of error. We identified the items and developed the SCARES score that correlated in a statistically significant manner with the risk of error. This score will need to be validated on a larger sample but this result can certainly indicate possible corrective interventions to reduce the risk of error, such as medication review, deprescribing, using child-friendly drugs and improving caregiver training and support.

Another important point is the caregiver’s perception of the medication regimen: 62% of them report that their children are taking too many medications and 23% are not completely satisfied with the symptoms. These data should be carefully analyzed trying to understand the psychological and social reasons at the base of this discontent, considering not only the clinical perspective. To note, 73% of caregivers responded positively to the possibility of having direct counseling with the clinical pharmacist to discuss their doubts about the therapy preparation or administration. Literature [30, 31] supports that pharmacist-led interventions can significantly reduce therapeutic errors and improve compliance rate but this has not been already evaluated in a PPC population.

To date, this is the first study that explores the impact of the management of domiciliary pharmacological therapy on a consistent population of caregivers in the context of PPC. The findings highlight how much time and effort caregivers need to go correctly through therapy preparation and administration. Also understanding their expectations and concerns is critical to indicate possible improvement actions. Lastly, once validated, the SCARES score, could be a simple tool to precociously identify risk factors for therapy delivery error.

After this study three action points have been activated in our center to reduce the burden on caregivers:

1) we organized systematic medication reviews of all the therapeutical regimens highlighted as difficult to manage by caregivers in a multidisciplinary team of clinicians and clinical pharmacists regularly;

2) we offered via tele-consultation the possibility for the caregivers to have direct contact with a clinical pharmacist to discuss further all the specificities of their medication regimen;

3) we implemented parental training done by our nurses on medication regimens with a pamphlet dedicated to the most common “daily challenges” in drug preparation as a reminder available on paper and at our center website [32, 33].

This study has a few limitations. First, this is a cross-sectional, non-prospective, and monocentric study. Furthermore, we developed an ad hoc questionnaire that was not validated as no standardized questionnaires are available to assess the impact of polypharmacy on caregivers in the context of PPC. For the SCARES score, we tested it in our population to assess its feasibility, but this is only the first step in the validation process which is crucial. Side effects or negative reactions related to CMR and off-label drug usage have not been studied, because this information was not the primary focus of our study. The possibility to repeat a more extended assessment and to validate our questionnaire and SCARES score will be the subject of future studies that will provide a more exhaustive characterization of medicine management among the PPC cohort.

## Conclusion

Many caregivers of Pediatric Palliative Care patients struggled to plan, prepare, and provide drug therapy to their children. Attempting to simplify medication regimens, selecting formulations that are simple to administer and measure, investing in improved caregiver training, discussing therapies with caregivers and involving clinical pharmacists in this process are all potential strategies to improve and reduce the burden of this condition.

## Electronic supplementary material

Below is the link to the electronic supplementary material.

Supplementary Material 1

## Data Availability

The datasets used and/or analyzed during the current study are available from the corresponding author on reasonable request.

## Abbreviations

ATC: Anatomical Therapeutic Chemical Classification
CMR: Complex Medication Regimen
DNR: Do Not Resuscitate
EC: Ethical Committee
G-tube: Gastrostomy tube
GCP: Good Clinical Practice
J-tube: Jejunostomy tube
LOS: Length Of Stay
NGT: Nasogastric Tube
PPC: Pediatric Palliative Care
QoL: Quality of Life
SCARES: Socio-Cultural Assessment for caregiver Risk of medication Error Score
SmPC: Summary of Product Characteristics
