# Supplementary material for: The role and perception of the caregiver in a specialized pediatric palliative care center in medicine preparation and administration: a survey study
Source: Ital J Pediatr. 2024 Nov 6;50:238. doi: 10.1186/s13052-024-01809-4 (PMC11539818; doi:10.1186/s13052-024-01809-4)
Supplement: Supplementary file 1 — Supplementary Material 1 [file 13052_2024_1809_MOESM1_ESM.docx]

Additional file 1. - Survey

**Area 1 (Medication Preparation)**

- Have you had any difficulties in the past three months in properly preparing medications for your child? (Example: problems with preparing oral suspensions, unable to open capsules, protective cap not opening, etc.)

□ Yes: __________________________________________________________

□ No

□ I don't remember

- Have you had any difficulties in the past three months in accurately measuring the dosage of your child's medications? (Example: the dosing dropper not fitting the bottle, tablets couldn't be split, etc.)

□ Yes

□ No

□ I don't remember

- How many times a day do you have to handle the medication to ensure proper administration to your child?

□ 1-2 times a day

□ 3-4 times a day

□ >5 times a day

□ Never

- On average, how much time is required for medication preparation during each administration?

□ Less than 1 minute

□ Between 1 and 5 minutes

□ Between 5 and 10 minutes

□ More than 10 minutes

- Have you received any training on properly preparing medications for your child, and if so, from whom?

□ Yes: __________________________________________________________

□ No

□ I don't remember

**Area 2 (Administration Management)**

- Have you ever had difficulties in the past in adhering to the timing interval for administering medications to your child? (Example: the child was sleeping, the child was not present, etc.)

□ Yes

□ I don't remember

- What is your preferred route of administration for your child's long-term medications?

□ Oral

□ Enteral route through PEG or NG tube

□ Transdermal route through the skin (e.g. patches, creams, etc.)

□ Rectal

□ Other __________________________________

- What is the reason for choosing this type of administration?

□ It takes less time for preparation

□ It doesn't require manipulation

□ It is well tolerated by my child

□ Other __________________________________

- Have you had any difficulties in the past three months with your child's acceptance during medication administration? (Example: the child reacted, the child spit everything out, etc.)

□ Always

□ Sometimes

□ Rarely

□ Never

□ I don't remember

- To facilitate administration, do you mix the medications with any food or beverage?

□ Yes

□ No

- If yes, how often?

□ Always

□ Occasionally

- What do you mix them with?

□ Water

□ Soft foods (yogurt/applesauce)

□ Fruit juice, milk, etc.

□ Special foods

□ Other: __________________________________

**Area 3 (Medication Error)**

- Have you ever administered medications incorrectly in the past three months?

□ Yes

□ No

□ I don't remember

- What do you think was the cause of this error?

□ I didn't feel adequately trained in medication preparation

□ I had too many administrations and got confused

□ I was tired and inattentive

□ Other: __________________________________

**Area 4 (Expectations)**

- Do you ever think your child takes too many medications?

□ Always

□ Often

□ Sometimes

□ Rarely

□ Never

- Are you overall satisfied with your child's current medication regimen?

□ Yes

□ Sometimes

□ Not entirely

□ Not at all

- Is there anything regarding your child's medications that you would like to discuss with the medical team?

□ Yes: _____________________________________

□ No

□ I don't know

- How do you evaluate the possibility of discussing the management of your child's medications with a dedicated pharmacist, either on a one-time or periodic basis, possibly remotely?

□ Very positively

□ Quite positively

□ Indifferent

□ Quite negatively

□ Negatively

□ I'm not interested because I rely on other health personnel

Additional file 2. - Principal symptoms and needs reported and their prevalence

|  | N (%) |
| --- | --- |
| **Neurological symptoms** | **89 (89%)** |
| Cognitive impairment | 74 (74%) |
| Mobility impairment | 55 (55%) |
| Seizures | 53 (53%) |
| Spasticity | 39 (39%) |
| Speech disorders | 35 (35%) |
| Hypotonia or asthenia | 32 (32%) |
| Sleeping disorders | 32 (32%) |
| Dystonia | 27 (27%) |
| Neuropathic pain | 22 (22%) |
| **Gastrointestinal symptoms** | **85 (85%)** |
| Dysphagia | 79 (79%) |
| Inadequate oral intake | 56 (56%) |
| Constipation | 44 (44%) |
| Drooling | 40 (40%) |
| Gastroesophageal reflux | 35 (35%) |
| Nausea or vomiting | 8 (8%) |
| **Respiratory symptoms** | **77 (77%)** |
| Excessive respiratory secretions | 46 (46%) |
| Chronic respiratory failure | 45 (45%) |
| Need of non-invasive ventilation | 23 (23%) |
| Need of mechanical ventilation | 9 (9%) |
| Apnea | 8 (8%) |
| **Musculoskeletal symptoms** | **75 (75%)** |
| Spinal deformity | 52 (52%) |
| Articular deformity | 49 (49%) |
| Musculoskeletal Pain | 27 (27%) |
| Osteoporotic fractures | 12 (12%) |
| **Sensory symptoms** | **50 (50%)** |
| Vision impairment | 44 (44%) |
| Hearing loss | 16 (16%) |
| **Nephrological symptoms** | **37 (37%)** |
| Urinary Dysfunction | 26 (26%) |
| Neurogenic Bladder | 5 (5%) |
| Urolithiasis | 3 (3%) |
| Vesicoureteral reflux | 3 (3%) |
| **Integumentary system symptoms** | **25 (25%)** |
| Pressure ulcer | 25 (25%) |
| Itching | 2 (2%) |
| Lymphedema | 2 (2%) |
| **Cardiocirculatory symptoms** | **16 (16%)** |
| Arrhythmia | 8 (8%) |
| Hypertension | 7 (7%) |
| Oedema | 2 (2%) |
| **Reproductive symptoms** | **15 (15%)** |
| Sexual needs | 7 (7%) |
| Dysmenorrhea | 3 (3%) |
| Painful Erections | 2 (2%) |

Additional file 3. Complete survey responses

|  | N (%) |
| --- | --- |
| Have you had any difficulties in the past three months in properly preparing medications for your child? (Example: problems with preparing oral suspensions, unable to open capsules, protective cap not opening, etc.) |  |
| Yes  No  I don't remember | 10%  88%  2% |
| Have you had any difficulties in the past three months in accurately measuring the dosage of your child's medications? (Example: the dosing dropper not fitting the bottle, tablets couldn't be split, etc.) |  |
| Yes  No  I don't remember | 15%  85%  None |
| How many times a day do you have to handle the medication to ensure proper administration to your child? |  |
| 1-2 times a day  3-4 times a day  >5 times a day  Never | 35%  42%  10%  13% |
| On average, how much time is required for medication preparation during each administration? |  |
| Less than 1 minute  Between 1 and 5 minutes  Between 5 and 10 minutes  More than 10 minutes | 19%  49%  19%  13% |
| Have you received any training on properly preparing medications for your child, and if so, from whom^1^? |  |
| Yes  No  I don't remember | 59%  41%  None |
| Have you ever had difficulties in the past in adhering to the timing interval for administering medications to your child? (Example: the child was sleeping, the child was not present, etc.) |  |
| Yes  No  I don't remember | 43%  57%  None |
| What is your preferred route of administration for your child's long-term medications? |  |
| Oral  Enteral route through PEG or NG tube  Transdermal route through the skin (e.g. patches, creams, etc.)  Rectal  Other | 35%  75%  None  None  None |
| What is the reason for choosing this type of administration? |  |
| It takes less time for preparation  It doesn't require manipulation  It is well tolerated by my child  Other: For patient’s underlying clinical condition | 2%  None  59%  39% |
| Have you had any difficulties in the past three months with your child's acceptance during medication administration? (Example: the child reacted, the child spit everything out, etc.) |  |
| Always  Sometimes  Rarely  Never  I don't remember | 4%  10%  11%  75%  None |
| To facilitate administration, do you mix the medications with any food or beverage? |  |
| Yes  No | 58%  42% |
| If yes, how often? |  |
| Always  Occasionally | 46/58 (79.3%)  12/58 (20.7%) |
| What do you mix them with? |  |
| Water  Soft foods (yogurt/applesauce)  Fruit juice, milk, etc.  Special foods  Other | 41/58 (70.7%)  7/58 (12.1%)  9/58 (15.5%)  1/58 (1.7%)  None |
| Have you ever administered medications incorrectly in the past three months? |  |
| Yes  No  I don't remember | 14%  83%  3% |
| What do you think was the cause of this error? |  |
| I didn't feel adequately trained in medication preparation  I had too many administrations and got confused  I was tired and inattentive  Other  Not reported | 1/14 (7.1%)  4/14 (28.6%)  5/14 (35.7%)  2/14 (14.3%)  2/14 (14.3%) |
| Do you ever think your child takes too many medications? |  |
| Always  Often  Sometimes  Rarely  Never | 29%  12%  14%  7%  38% |
| Are you overall satisfied with your child's current medication regimen? |  |
| Yes  Sometimes  Not entirely  Not at all | 77%  8%  15  None |
| Is there anything regarding your child's medications that you would like to discuss with the medical team? |  |
| Yes  No  I don't know | 39%  59%  2% |
| How do you evaluate the possibility of discussing the management of your child's medications with a dedicated pharmacist, either on a one-time or periodic basis, possibly remotely? |  |
| Very positively  Quite positively  Indifferent  Quite negatively  Negatively  I'm not interested because I rely on other health personnel | 58%  15%  15%  1%  2%  9% |
